# Editorial: Metabolic alterations in cancer: from targeting metabolism to cancer detection

**DOI:** 10.3389/fcell.2025.1661985

**Published:** 2025-07-30

**Authors:** Tobias Ackermann, Ruhi Deshmukh, Cesar Echeverria, Juan F. Santibanez, Victor H. Villar

**Affiliations:** ^1^ Laboratory for Experimental Oncology and Radiobiology, Cancer Center Amsterdam, Amsterdam UMC, University of Amsterdam, Amsterdam, Netherlands; ^2^ Oncode Institute, Amsterdam, Netherlands; ^3^ Cancer Research UK Scotland Institute, Glasgow, United Kingdom; ^4^ Instituto de Ciencias Naturales, Facultad de Medicina Veterinaria y Agronomía, Universidad de las Américas, Santiago, Providencia, Chile; ^5^ Centro de investigación en Ciencias Biológica y química, Universidad de las Américas, Santiago, Chile; ^6^ Institute for Medical Research, National Institute of the Republic of Serbia, University of Belgrade, Belgrade, Serbia; ^7^ School of Medicine, Mackenzie Institute for Early Diagnosis, University of St Andrews, St Andrews, United Kingdom

**Keywords:** mitophagy, lung adenocarcinoma, glioblatoma, ovarian cancer, cancer metabolism

One of the most fundamental hallmarks of cancer cells is their uncontrolled proliferation. While this increased proliferative capacity is primarily driven by genetic alterations in oncogenes and tumour suppressor genes, it ultimately relies on metabolic reprogramming to support the generation of biomass and energy. These genetic alterations lead to specific reorganization of cellular metabolism, creating vulnerabilities that can be exploited to develop or identify better therapies against cancer.

In this Research Topic, the metabolic alterations associated with cancer, their potential as therapeutic targets, and their use in patient stratification are discussed in the context of glioblastoma (GB), ovarian cancer (OC), and lung adenocarcinoma (LA). In addition, autophagy-dependent mitochondrial degradation, also known as mitophagy, is discussed in the context of tumour biology and its potential as a target for future anti-cancer therapies.

Mitochondria are crucial metabolic organelles where many fundamental metabolic reactions occur, including oxidative phosphorylation, nucleotide biosynthesis, TCA cycle, among others. Consequently, the process responsible for removing damaged mitochondria, known as mitophagy, has been linked with cancer progression and cell survival. Dong and Zhang in their review provide insights into the role of mitophagy in cancer, focusing on its therapeutic potential across many different cancer types. The authors discuss the molecular mechanisms underlying mitophagy, including the PINK1/Parkin pathway and the non-ubiquitin-dependent pathway such as BNIP3/NIX. Authors also explore the role of mitochondria dynamics, fusion and fission, in the regulation of mitophagy. Furthermore, the review addresses the role of mitophagy in many aspects of cancer biology, such as tumour progression, metastasis, tumour resistance (including immunotherapy and radiotherapy), stemness, cancer metabolism, and ferroptosis. Authors also describe the role of mitophagy in other cell types present in the tumour microenvironment, such as cancer-associated fibroblasts. In summary, the authors provide strong evidence that mitophagy is involved in many aspects of cancer biology, highlighting that further research, and understanding will enable mitophagy to become a therapeutic target across different cancer types.

Ferroptosis, a form of regulated cell death distinct from apoptosis, has gained attention for its prospective in targeting cancer cells resistant to traditional therapies. Wu et al. describe the potential of targeting ferroptosis in OC treatment. The review outlines the molecular pathways controlling ferroptosis in OC, highlighting key regulators such as SLC7A11, GPX4, and ACSL4. It emphasizes how the dysregulation of these pathways can lead to lipid peroxidation and iron accumulation, essential elements of ferroptotic cell death. Moreover, the review explores the complex network of microRNAs, long non-coding RNAs, and circular RNAs that modulate ferroptosis sensitivity, offering insights into potential biomarkers for therapeutic targeting. Therapeutically, the review discusses various strategies to induce ferroptosis in OC cells, including the use of small molecule drugs, gene editing tools, and natural products. These approaches aim to overcome the limitations of conventional treatments like chemotherapy, which often face challenges due to tumour heterogeneity and drug resistance. In conclusion, the review underscores the therapeutic potential of ferroptosis in OC, advocating for further research to translate these findings into clinical applications. By targeting ferroptotic pathways, there is hope for developing more effective treatments for OC patients, particularly those with refractory disease.

D’Aprile et al., review the reprogramming of various metabolic pathways that occurs in GB. This heterogeneous disease is characterised by alterations in the bioenergetic pathways, including glucose, lipid, and amino acid metabolism, thus impacting nucleotide and iron metabolic activity. Although these changes support tumour progression and may drive resistance to therapies, they also present certain vulnerabilities, which can be exploited as therapeutic targets against this deadly disease. The authors summarise such strategies that are currently under clinical investigation. Targeting the reprogrammed metabolism in GB can serve as an adjuvant to the conventional radiotherapy and chemotherapy, which the authors suggest warrants further studies, especially considering the heterogeneity of the disease.

Polyamines are small polycationic molecules including putrescine, spermidine, and spermine. These small metabolites are essential for cell growth, DNA replication, proliferation and apoptosis. Li et al. described in their article that the levels of three proteins associated with the polyamine metabolism, proteasome 26S subunit ATPase 6 (PSMC6), spermine oxidase (SMOX), and spermine synthase (SMS) in LA can be used to generate a polyamine metabolism-related score (PMRS). Tumour tissue showed a higher PMRS compared with normal healthy tissue. In addition, the PMRS has prognostic value, as a high score is associated with poor outcomes in LA and can also aid in predicting the sensitivity to immunotherapy and chemotherapy.

This Research Topic hopes to highlight that identifying specific metabolic alterations or regulatory processes, such as mitophagy, have a strong potential not only for the identification of novel therapeutic targets but also for developing tools to predict treatment outcome. Altogether this could aid clinicians in identifying and selecting the best therapeutical option to improve the survival of cancer patients.

